# Coagulation biomarkers and coronavirus disease 2019 phenotyping: a prospective cohort study

**DOI:** 10.1186/s12959-023-00524-0

**Published:** 2023-07-28

**Authors:** Emily Corneo, Rafael Garbelotto, Gabriele Prestes, Carolina Saibro Girardi, Lucas Santos, Jose Claudio Fonseca Moreira, Daniel Pens Gelain, Glauco A. Westphal, Emil Kupek, Roger Walz, Cristiane Ritter, Felipe Dal-Pizzol

**Affiliations:** 1 Laboratory of Experimental Pathophysiology, Graduate Program in Health Sciences, University of Southern Santa Catarina (UNESC), Santa Catarina, Av. Universitária, Criciúma, 1105 Brazil; 2 Intensive Care Unit, Hospital São José, Criciúma, SC Brazil; 3 Departamento de Bioquímica, Centro de Estudos Em Estresse Oxidativo, Instituto de Ciências Básicas da Saúde, Universidade Federal Do Rio Grande Do Sul, Porto Alegre, RS Brazil; 4 Centro Hospitalar Unimed, Joinville, SC Brazil; 5 Public Health Department, Federal University of Santa Catarina, Florianópolis, SC Brazil; 6 Center for Applied Neuroscience (CeNAp), Department of Clinical Medicine, University Hospital - Federal University of Santa Catarina, Florianópolis, SC Brazil; 7 Clinical Research Center, Hospital São José, Criciúma, SC Brazil

**Keywords:** COVID-19, Coagulation, Coagulation factors, Cluster phenotyping, Prognostication

## Abstract

**Background:**

Because severe acute respiratory syndrome coronarivus 2 (SARS-CoV-2) leads to severe conditions and thrombus formation, evaluation of the coagulation markers is important in determining the prognosis and phenotyping of patients with COVID-19.

**Methods:**

In a prospective study that included 213 COVID-19 patients admitted to the intensive care unit (ICU) the levels of antithrombin, C-reactive protein (CRP); factors XI, XII, XIII; prothrombin and D-dimer were measured. Spearman’s correlation coefficient was used to assess the pairwise correlations between the biomarkers. Hierarchical and non-hierarchical cluster analysis was performed using the levels of biomarkers to identify patients´ phenotypes. Multivariate binary regression was used to determine the association of the patient´s outcome with clinical variables and biomarker levels.

**Results:**

The levels of factors XI and XIII were significantly higher in patients with less severe COVID-19, while factor XIII and antithrombin levels were significantly associated with mortality. These coagulation biomarkers were associated with the in-hospital survival of COVID-19 patients over and above the core clinical factors on admission. Hierarchical cluster analysis showed a cluster between factor XIII and antithrombin, and this hierarchical cluster was extended to CRP in the next step. Furthermore, a non-hierarchical K-means cluster analysis was performed, and two phenotypes were identified based on the CRP and antithrombin levels independently of clinical variables and were associated with mortality.

**Conclusion:**

Coagulation biomarkers were associated with in-hospital survival of COVID-19 patients. Lower levels of factors XI, XII and XIII and prothrombin were associated with disease severity, while higher levels of both CRP and antithrombin clustered with worse prognosis. These results suggest the role of coagulation abnormalities in the development of COVID-19 and open the perspective of identifying subgroups of patients who would benefit more from interventions focused on regulating coagulation.

**Supplementary Information:**

The online version contains supplementary material available at 10.1186/s12959-023-00524-0.

## Background

According to disease severity, excessive production of thrombin, damage to the endothelial cells, inhibition of fibrinolysis, activation of the complement pathway, deposition of microthrombi and microvascular dysfunction, are characteristics of COVID-19 patients [1–4]. Patients admitted to the hospital due to severe COVID-19 are predisposed to endothelial cell activation and injury, platelet activation, and hypercoagulability. One of the consequences of COVID-19 is thrombotic and thromboembolic events, such as disseminated intravascular coagulation, deep vein thrombosis and pulmonary embolism [5, 6]. Arterial thrombosis, including ischemic stroke, acute coronary syndrome, limb ischemia and systemic arterial embolism can occur. The incidence varies according to the severity of the disease, with a higher prevalence in patients admitted to the intensive care unit (ICU) [7, 8].

Excessive inflammation caused by SARS-CoV-2 systemically activates blood clotting, probably due to the release of von Willebrand factor (vWF) and increased endothelial cell surface expression of adhesion molecules, favoring thrombus formation [9–11]. Consequently, the activation of the coagulation cascade promotes platelet aggregation, neutrophil activation and the release of neutrophil extracellular traps that propagate vascular and organ injury [9–14].

Elevated levels of clotting markers are important for determining the prognosis of patients with COVID-19, as infection with SARS-CoV-2 can lead to severe conditions and thrombus formation [6, 15]. Assessment of blood clotting factors, including pro-clotting factors such as fibrinogen, prothrombin, and factors XI, XII, and XIII, and natural anticoagulants such as antithrombin is critical in understanding the pathophysiological processes underlying the development of COVID-19 and its complications. Furthermore, analyzing these markers can help identify individuals at an increased risk of developing thrombosis and potentially guide thromboprophylaxis and treatment approaches [16, 17].

Patients with COVID-19 have high D-dimer levels, prolonged prothrombin time (PT) or activated partial thromboplastin time (aPTT), decreased factor V activity, and hypofibrinogenemia [18–21]. A recent meta-analysis on coagulation dysfunction found that the D-dimer levels, fibrinogen levels, aPTT and PT were significantly higher in severe COVID-19 patients [22]. However, most of the included studies were retrospective and did not consider the potential confounding factors affecting the association between coagulation markers and disease severity. In addition, data on the coagulation tests that are not routinely used in clinical practice are lacking. In this context, the prognostic value of coagulation biomarkers (CB) over and above the disease severity scores already in use has gained importance in prioritizing patient care.

The present prospective cohort study of hospitalized COVID-19 patients aimed to verify the following hypotheses: a) patient clustering of non-conventional coagulation parameters is predictive of in-hospital survival, and b) these biomarkers can be used in combination with conventional clinical parameters in the prognostication of in-hospital survival.

## Methods

### Study design

A prospective cohort study was conducted on patients admitted to two tertiary hospitals in southern Brazil between June 2020 and November 2020. The study was approved by the Institutional Review Board of each institution. In addition, all patients or their surrogates provided written informed consent before their inclusion in the study.

### Setting

The study sample consisted of all consecutive patients admitted to the intensive care unit (ICU) of participating hospitals from June 2020 to November 2020.

### Participants

Patients aged > 18 years who were diagnosed with COVID-19 through reverse transcriptase reaction or rapid antigen test and required supplementary oxygen (World Health Organization (WHO) class 4), noninvasive ventilation (WHO class 5), or invasive mechanical ventilation (WHO class 6) due to COVID-19 pneumonia were included in the study. By contrast, patients with severe chronic diseases (chronic kidney disease undergoing dialysis, Child–Pugh class C cirrhosis, severe chronic obstructive pulmonary disease, severe heart failure) or diseases that alter the inflammatory response, such as those with long-term use of immunosuppressants, with cancer without disease control, with human immunodeficiency virus infection without disease control, and who received palliative care, or with a life expectancy of less than 24 h as judged by the attending physician were excluded.

### Procedures

After patient inclusion, venous blood samples were collected within 24 h after ICU admission; meanwhile the sociodemographic and clinical information was collected directly from the patient, their surrogate, or the electronic medical records. The levels of CB (antithrombin; C-reactive protein (CRP); factors XI, XII, and XIII; and prothrombin) were measured using the Coagulation 6-Plex Human ProcartaPlex Panel 1 (Cat. #EPX060-10824–901), from Thermo Fisher Scientific, (Waltham, MA, USA) in a Luminex MAGPIX® system (Luminex Corporation -Austin, TX, USA). Final protein concentrations were calculated using the online Procarta Plex Analysis Application (Thermo Fisher Scientific) and expressed as arbitrary units from the reference plasma. Additionally, as a conventional coagulation biomarker D-dimer was measured using an ELISA kit according to manufacturer instructions (Thermo Fisher Scientific, Waltham, MA, USA).

### Statistical analysis

The independent groups were compared using a Mann–Whitney U test or a Kruskal–Wallis test and chi-square test for continuous and categorical variables in the univariate analyses. Spearman’s correlation coefficient was used to assess the pairwise correlations between the numerical variables. A hierarchical cluster of the corresponding correlation matrix was performed to identify the clusters of co-expressed biomarkers. Additionally, a non-hierarchical* K*-means clustering analysis was performed to assess for CB clustering, which was subsequently related to the outcome. Multivariate binary (logistic) regression was used to determine the association of the patient´s outcome with other independent variables, conceptually divided into core demographic (age and sex) and clinical prognostic factors (SAPS III, Charlson score, chest computed tomography (CT) score, and body mass index (BMI)), hitherto denominated “core predictors”, and the aforementioned CB. The latter were categorized into quintiles owing to their highly non-normal distributions, with missing values fitted as an additional category to avoid the impact of selective dropout. The receiver operating curves (ROC) for core alone and core plus the CB predictors were compared in terms of accuracy, measured by the area under the ROC (AUROC), as well as the sensitivity, specificity, positive and negative predictive values, and likelihood ratios of positive and negative tests. The AUROC was cross-validated on five independent samples to avoid model fitting and evaluation of the same sample. Bootstrap bias corrected 95% confidence intervals (CI) were used to express the AUROC uncertainty.

Data were analyzed using IBM SPSS Statistics version 22.0 (IBM Corp., Armonk, N.Y., USA) and Stata version 13.1 (StataCorp, College Station, TX, USA) software. The type I error level was set to 0.05 in all statistical analyses.

## Results

### Clinical characteristics of the sample

The demographic data, comorbidities, disease characteristics at hospital admission and clinical outcomes of the 213 patients in the analytical sample are listed in Table 1. The in-hospital mortality in the analytical sample was 25% (53 of 213 patients). Results of the univariate analysis showed that age, need for mechanical ventilation, comorbidities (measured based on the Charlson score), BMI, disease severity at ICU admission (measured based on the SAPS III score), and degree of organ dysfunction (measured based on the total SOFA score at admission), were all associated with mortality.

**Table 1:** Clinical characteristics of included patients

	Survivor (*n* = 160)	Non-Survivor (*n* = 53)	*p*-value^*^
Gender, male, n (%)	61 (38)	16 (30)	0.32
Age, mean (SD)	53 (15)	62 (13)	< 0.001
Need for mechanical ventilation, n (%)	46 (29)	37 (70)	< 0.001
Thorax CT scan extension of lesions > 50%, n (%)	51 (32)	28 (53)	0.023
Charlson comorbidity index, mean (SD)	1.62 (1.5)	2.34 (1.65)	0.004
BMI, mean (SD)	26 (10)	29 (5)	0.026
SOFA at admission, mean (SD)	2.78 (2.2)	5.28 (3.6)	< 0.001
SAPS III score, mean (SD)	39 (25)	58 (20)	< 0.001
Respiratory SOFA, mean (SD)	1.98 (1)	2.43 (1)	0.009

^*^Hypothesis of no difference between alive and dead

*BMI* Body mass index;

*CT* Computed tomography

*SAPS* Simplified Acute Physiologic Score

*SD* Standard Deviation;

*SOFA* Sequential Organ Failure Assessment

*%* percentage of the subgroup total

### Coagulation parameters and disease severity

**Fig. 1:**
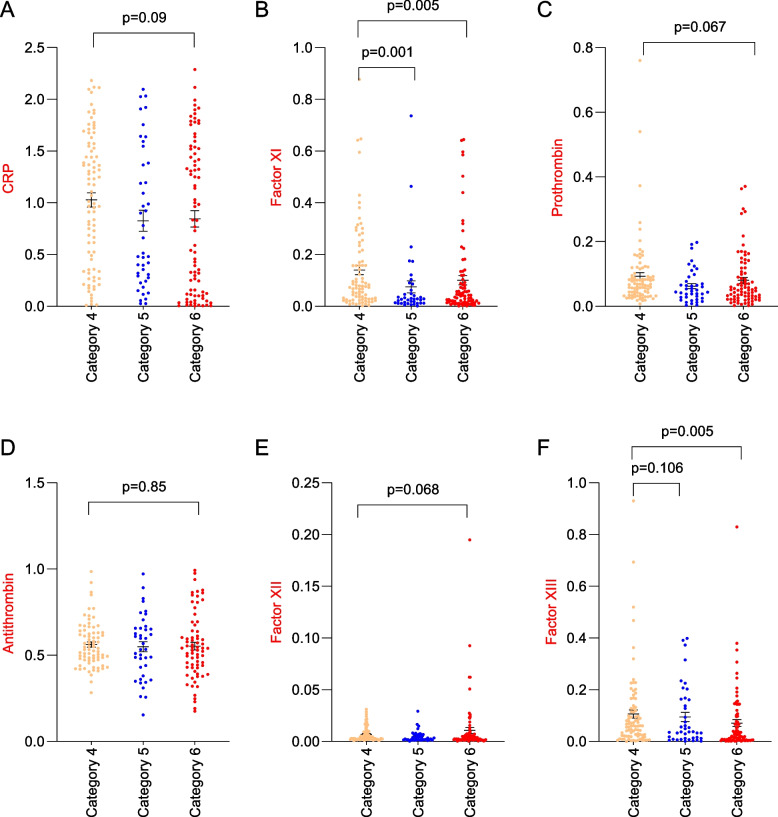
Coagulation biomarkers and COVID-19 severity

**Fig. 2:**
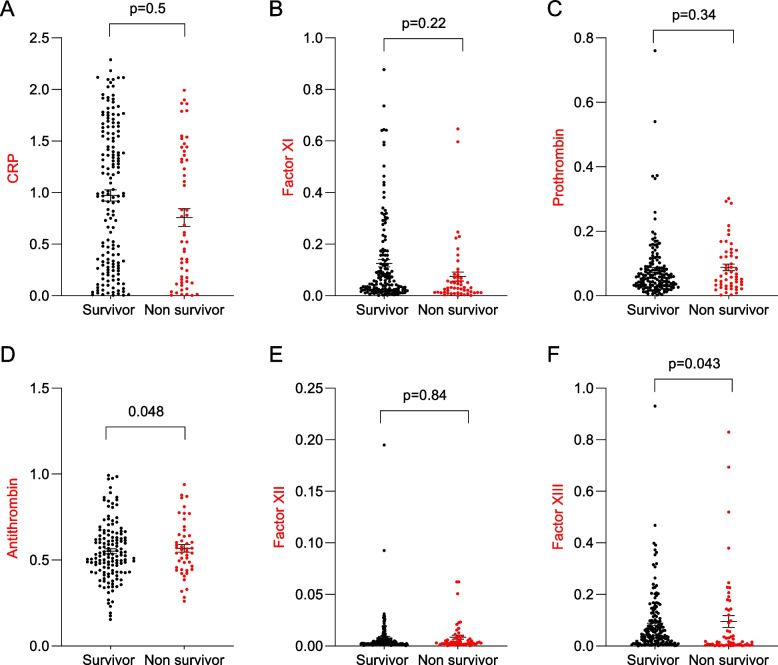
Coagulation biomarkers and COVID-19 mortality

First, the differences in the levels of measured CB between the three crescent WHO ordinal scale severities (from 4 to 6, Fig. 1 A-F). Only the concentrations of factors XI (Fig. 1B) and XIII (Fig. 1F) were significantly different between the groups (*p* = 0.01 and *p* = 0.013, respectively), being higher in those with less severe types of COVID-19. The levels of prothrombin (Fig. 1C) and factor XII (Fig. 1E) were higher in the WHO category 4 patients. D-dimer levels did not differ between groups (*p* = 0.55). Additionally, the levels of antithrombin (Fig. 2D) and factor XIII (Fig. 2F) were significantly associated with mortality. Furthermore, D-dimer levels were also associated with mortality (453 ± 308 *vs* 704 ± 470, *p* = 0.0001, in survivors and non-survivors respectively).

**Fig. 3:**
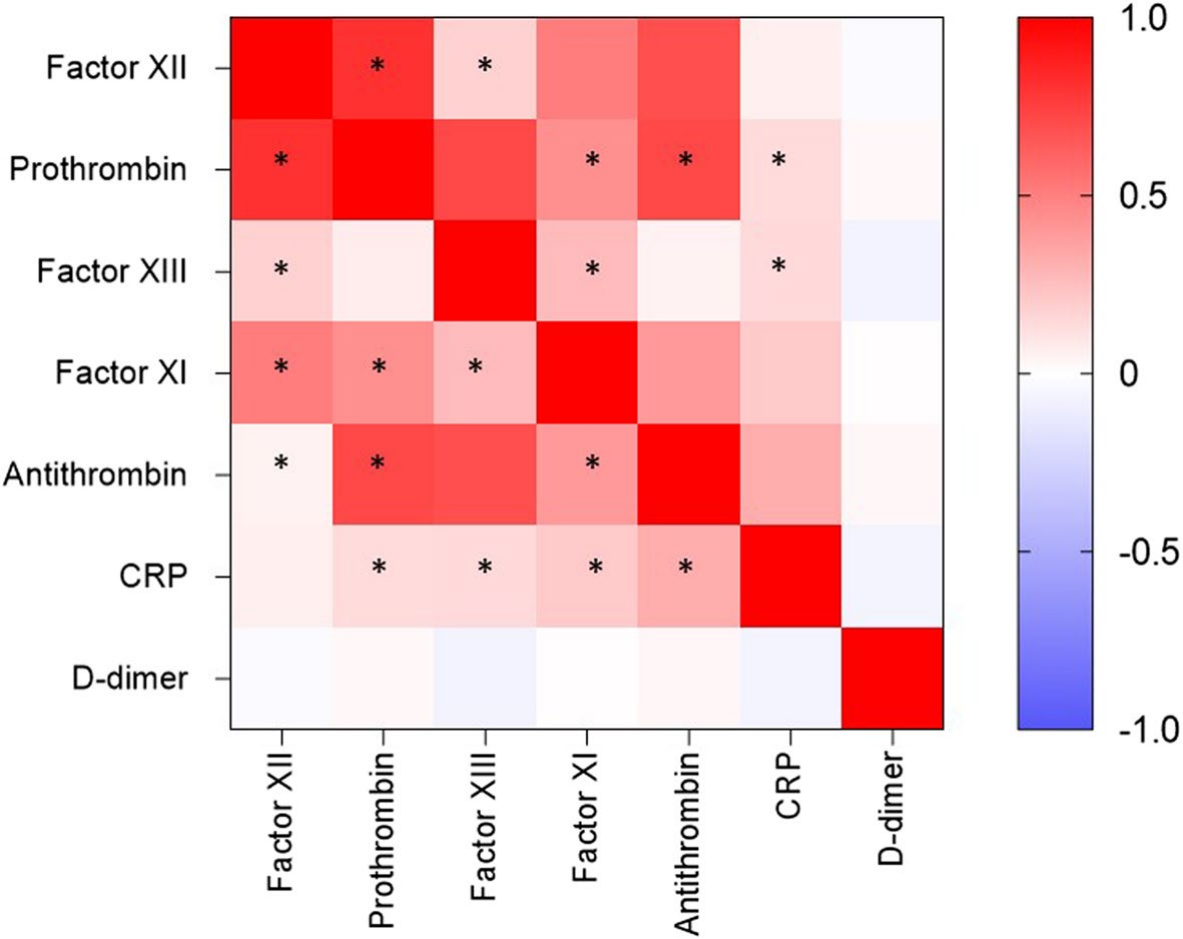
Spearman´s correlations between biomarker levels. The magnitude of each correlation is denoted with a colour, whereby the red colour indicates a positive correlation, and the blue colour indicates a negative correlation. * denotes significant correlation (*p* < 0.05)

Spearman’s correlation coefficient was used to assess the pairwise correlation of these biomarkers (Fig. 3). Differently from non-conventional CB, D-dimer did not significantly correlate to any of another measured parameter (Fig. 3). Using hierarchical clusters it was observed a cluster between factor XIII and antithrombin reinforced the observed association between factor XIII and antithrombin levels and in-hospital mortality. Interestingly, this hierarchical cluster was extended to CRP. To further explore a potential relationship, a non-hierarchical K-means cluster analysis was performed. Two phenotypes were identified based on the CRP and antithrombin levels, but they did not include factor XIII (Table 1, Supplementary Material). Despite the higher mortality rate among phenotype 2 patients compared with phenotype 1 patients, their clinical characteristics were similar, except for the higher total organ dysfunction score (Table 2). D-dimer did not significatively add to non-hierarchical K-means cluster. Adding D-dimer to the other biomarkers phenotype 1 included only 2 patients.

**Table 2:** Biomarkers phenotyping and clinical characteristics

	Phenotype 1 (*n* = 95)	Phenotype 2 (*n* = 92)	*p*-value^*^
Gender, male, n (%)	65 (68)	56 (61)	0.28
Age, mean (SD)	54 (16)	56 (14)	0.13
Need for mechanical ventilation, n (%)	32 (34)	38 (41)	0.28
Thorax CT scan extension of lesions > 50%, n (%)	36 (38)	34 (37)	0.88
Charlson comorbidity index, mean (SD)	1.72 (1.5)	1.72 (1.6)	0.50
BMI, mean (SD)	27 (10)	27 (8)	0.40
SOFA at admission, mean (SD)	2.98 (2.4)	3.79 (3.1)	0.02
SAPS III score, mean (SD)	41 (25)	47 (24)	0.11
Respiratory SOFA, mean (SD)	2.04 (1.1)	2.17 (1.0)	0.19
Non-survivor, n (%)	18 (19)	30 (33)	0.03

^*^Hypothesis of no difference between the phenotypes

*BMI* Body mass index;

*CT* Computed tomography

*SAPS* Simplified Acute Physiologic Score

*SD* Standard Deviation;

*SOFA* Sequential Organ Failure Assessment

*%* percentage of the subgroup total

We further investigated the diagnostic performance of the CB in the multivariate logistic regression analysis of in-hospital death (Table 2, Supplementary Material). Adding the CB to the core clinical predictors (Table 3) significantly improved the AUROC (*p* = 0.036), from 79% (95% CI 73%—84%) to 86% (95% CI 81%—90%). Significant improvements were also observed in the sensitivity value (from 38 to 57% (*p* = 0.02), positive predictive value (PPV) (from 65 to 81% (*p* = 0.054), and for likelihood ratios of a positive/negative test (*p* < 0.001). A two-fold increase in the likelihood ratio of a positive test following the addition of the biomarkers (Table 3) suggests a two-fold increase in the odds of correctly predicting in-hospital mortality. Adding D-dimer to core clinical predictors also improved the AUROC (from 79%—95% CI 73% to 84% *vs* 80%—95% CI 72% to 88).

**Table 3:** Diagnostic parameters comparison of two in-hospital survival models derived by multivariate logistic regression and fivefold cross-validation

Parameter name	**Clinical predictors**			**Clinical + CB**			Parameter difference *p*-value
	value	95% CI bounds		value	95% CI bounds		
		lower	upper		lower	upper	
Sensitivity (%)	38	26	51	57	43	69	0.020
Specificity	93	88	96	96	91	98	0.173
PPV (%)	65	47	79	81	66	91	0.054
NPV (%)	82	76	87	87	81	91	0.093
AUROC (%)	79	73	84	86	81	90	0.036
LRT +	5.5	3.9	7.7	13	9.3	18	< 0.001
LRT-	0.67	0.63	0.71	0.45	0.42	0.49	< 0.001

CB are the coagulation biomarkers

LRT + / LRT- are Likelihood Ratios of a positive/negative test;

PPV/NPV are positive / negative predictive values; AUROC is the area under ROC curve

If the false positive test result ranges from 10–20%, which is considered acceptable in several screening applications, the addition of CB increases the chance of correctly predicting the in-hospital death among COVID-19 patients by approximately 20%-30% compared with the ROC without the CB (Fig. 1, Supplementary Material).

## Discussion

Here, a significant improvement was observed in predicting COVID-19 in-hospital mortality when non-conventional CB were added to the core clinical indicators of disease severity assessed on ICU admission. The significant improvements in various diagnostic parameters, such as sensitivity, PPV, accuracy, and diagnostic odds, indicate the relevant gains in various aspects of predicting in-hospital death. Additionally, two different biomarkers were associated with mortality, and were clustered based on CRP and antithrombin levels. Conventional coagulation parameters, such as D-dimer, were also associated with mortality, but surprisingly did not help to clustering patients in phenotypes.

From the beginning of the pandemic, blood coagulation, assessed based on the D-dimer levels, has been associated with higher mortality rates [23]. Additional data suggest that several other routinely assessed coagulation parameters, such as platelet count, PT, and fibrinogen, were associated with disease severity and mortality [24]. From a mechanistic point of view, hypercoagulability is associated with elevated vWF, endothelial dysfunction, elevated fibrinogen and factor VIII levels, and reduced thrombomodulin levels [25]. Using a similar approach, Ceballos et al. 2021 [16] found a significant reduction in factor XI, factor XII, and factor XIII levels in patients with severe COVID-19. Herein, the levels of factors XI and XIII were statistically higher in the WHO category 4 group than in the more severe forms group. The levels of factor XII (*p* = 0.068) and prothrombin (*p* = 0.067) showed a trend to be higher, reinforcing the possible role of a procoagulant state in the progression of COVID-19. However, Ceballos et al. 2021 [16] study has several limitations. For example, noninvasive ventilated patients were not mentioned; blood samples were collected within a median time of 2 days (IQR 4 days). Additionally, Ceballos et al. [16] divided the patients into three quantiles (low, medium and high protein levels) and reported lower levels of coagulant factors in non-survivors; however, adding coagulation proteins to the survival models that included sex and age did not improve the survival prediction. In our study, categorizing CB into quintiles led to an improvement in prognostic assessment compared with the use of clinical variables alone. Furthermore, the sensitivity increased in the region with a low (< 20%) false-positive rate, which may be useful for prioritizing ICU resources.

Another relevant aspect of coagulation disorders and COVID-19 is whether a specific treatment aimed at coagulation would benefit the patients. Early in the pandemic, some authors recommended stratifying doses according to the D-dimer levels or extended post-discharge thromboprophylaxis [26]. Existing evidence shows that therapeutic-dose heparin benefits non-critically ill patients hospitalized due to COVID-19 [27–29], but it could induce harm in the critically ill [27]. Thus, the characteristics of patients who would benefit more from anticoagulation therapy should be indicated. Both hierarchical and k-cluster analyses could be used to stratify patients based on CB. In the k-cluster analyses, one phenotype was associated with the most severe organ dysfunction and higher mortality rates. It was not expected that D-dimer did not improve patients´ phenotyping. This finding opens the perspective that different phenotypes would respond differently to anticoagulation treatments, and further studies should address this issue. Using creatinine, albumin, CRP, white blood cell count and clinical characteristics five phenotypes of hospitalized COVID-19 patients were identified [30]. Patients with one of these phenotypes had renal failure, hypoalbuminemia, anemia, lymphopenia, and elevated CRP level (median, 9.0 mg/dL), and had the highest likelihood of ICU transfer or in-hospital mortality (59%). The use of 22 candidate variables for clustering analysis that included demographic information, disease history, major clinical symptoms, and medications on the day of positive diagnosis could also help determine the phenotype [31]. In these patients, three sub-phenotypes, determined mainly by a history of chronic hypertension, the presence of fever, development of respiratory and non-respiratory symptoms, and age, were associated with clinical deterioration. Recently, using four distinct clinical phenotypes described in non-COVID-19 septic patients it was determined that some of these phenotypes were more common in bacterial, when compared to viral sepsis [32]. Additionally, some phenotypes were associated with better outcomes after the introduction of dexamethasone therapy in COVID-19, reinforcing the idea that phenotyping patients would impact on prognostication and treatment stratification. To the best of our knowledge the present study was the first to provide discriminative phenotypes based only on the levels of CB.

This study has some limitations. First, the data were collected only from two hospitals in South Brazil, thus multicenter studies are needed to verify the generalizability of the study findings. Nevertheless, many patients with sufficient outcomes (severity and mortality) were included to allow robust regression and cluster analyses. Second, only a restricted number of coagulation proteins were analyzed at a single time point after hospital admission, leading to the lack of information on coagulation modifications over time and on more conventional coagulation parameters. However, several studies were conducted in the latter [24], hence our findings significantly add to the literature. Third, all included patients were not vaccinated, thus it is not possible to ascertain how the post-vaccination status impacts these results. Some data support that clinically these patients are similar regarding clinical characteristics, at least when hospitalization is needed [33]. Not only the vaccination status, but reinfection could also interfere in the immune response, and consequently in biomarker profile. For example, increased risks of haematological and vascular events that led to hospital admission or death were observed for short time intervals after first doses of vaccines. The risks of most of these events were substantially higher and more prolonged after SARS-CoV-2 infection than after vaccination in the same population [34]. Thus, coagulation biomarkers or coagulation-related events could be potentiated in vaccinated patients that develop severe COVID-19. Fourth, we did not measure coagulation biomarkers in healthy individuals, and this could add some valuable information to our data interpretation.

## Conclusion

CB were associated with the in-hospital survival of COVID-19 patients over and above the core clinical factors on admission. Adding these biomarkers significantly increased the sensitivity, PPV, accuracy, and diagnostic odds. Lower levels of some blood clotting factors were associated with disease severity, while higher levels of both CRP and antithrombin clustered with worse prognosis. These results suggest the role of coagulation abnormalities in COVID-19 development and help to identify the subgroups of patients who would benefit more from interventions focused on coagulation.

## Supplementary Information

**Additional file 1.**

## Data Availability

The datasets used and/or analysed during the current study are available from the corresponding author on reasonable request.

## Abbreviations

aPTT: Activated partial thromboplastin time
AUROC: Area under receiver operating curve
BMI: Body mass index
CB: Coagulation biomarkers
CT: Computed tomography
COVID-19: Coronavirus disease 2019
CRP: C-reactive protein
ICU: Intensive care unit
PPV: Positive predictive value
PT: Prothrombin time
SOFA: Sequential Organ Failure Assessment
SARS-CoV-2: Severe acute respiratory syndrome coronarivus 2
SAPS: Simplified Acute Physiology Score
vWF: Von Willebrand factor
WHO: World Health Organization
