# Supplementary material for: Coagulation biomarkers and coronavirus disease 2019 phenotyping: a prospective cohort study
Source: Thromb J. 2023 Jul 28;21:80. doi: 10.1186/s12959-023-00524-0 (PMC10375602; doi:10.1186/s12959-023-00524-0)
Supplement: Supplementary file 1 — Additional file 1. [file 12959_2023_524_MOESM1_ESM.docx]

Supplement: Table 1 – K-means cluster ANOVA results

|  | F-value | p-value |
| --- | --- | --- |
| CRP | 664.3 | <0.0001 |
| Antithrombin | 6.61 | 0.011 |
| Factor XI | 0.19 | 0.732 |
| Factor XII | 1.87 | 0.174 |
| Prothrombin | 2.30 | 0.131 |
| Factor XIII | 0.32 | 0.568 |

CRP – c-reactive protein

Supplement: Table 2 - Multivariate logistic regression with clinical and coagulation biomarker predictors of in-hospital death among COVID-19 patients

| **Patient**  **characteristics** | Level | OR | 95% Confidence limits | | p-value |
| --- | --- | --- | --- | --- | --- |
|  |  |  | Lower | upper |  |
| **Clinical** | | | | | |
| Simplified Acute Physiologic Score (SAPS) | 0-30 | 1.00 |  |  |  |
|  | 31-50 | 3.14 | 0.48 | 20.59 | 0.233 |
|  | 51-70 | 4.43 | 0.58 | 33.70 | 0.150 |
|  | 71+ | 17.32 | 2.25 | 133.03 | 0.006 |
|  | missing | 3.52 | 0.54 | 22.96 | 0.188 |
| Gender | Male | 1.00 |  |  |  |
|  | Female | 0.30 | 0.10 | 0.86 | 0.025 |
| Age (years) |  | 1.04 | 0.99 | 1.09 | 0.106 |
| BMI |  | 1.05 | 0.98 | 1.12 | 0.159 |
| SOFA (total on admission) |  | 1.21 | 1.01 | 1.45 | 0.044 |
| Charlson comorbidity index |  | 0.88 | 0.58 | 1.33 | 0.540 |
| Thorax CT scan extension of lesions | 25-50% | 1.00 |  |  |  |
|  | 50-75% | 0.40 | 0.10 | 1.55 | 0.186 |
|  | >75% | 0.99 | 0.29 | 3.36 | 0.991 |
| **Biomarker quintiles** | | | | | |
| C-reactive protein | 1^st^ | 1.00 |  |  |  |
|  | 2^nd^ | 0.42 | 0.10 | 1.83 | 0.246 |
|  | 3^rd^ | 0.25 | 0.06 | 1.07 | 0.062 |
|  | 4^th^ | 0.39 | 0.08 | 1.89 | 0.244 |
|  | 5^th^ | 0.27 | 0.05 | 1.41 | 0.120 |
| Antithrombin | 1^st^ | 1.00 |  |  |  |
|  | 2^nd^ | 3.43 | 0.61 | 19.38 | 0.164 |
|  | 3^rd^ | 3.76 | 0.59 | 23.87 | 0.160 |
|  | 4^th^ | 2.79 | 0.42 | 18.53 | 0.288 |
|  | 5^th^ | 5.53 | 0.66 | 46.69 | 0.116 |
|  | missing | 2.31 | 0.28 | 19.20 | 0.438 |
| Factor XI | 1^st^ | 1.00 |  |  |  |
|  | 2^nd^ | 0.51 | 0.11 | 2.42 | 0.398 |
|  | 3^rd^ | 0.31 | 0.06 | 1.67 | 0.173 |
|  | 4^th^ | 0.19 | 0.03 | 1.12 | 0.067 |
|  | 5^th^ | 0.17 | 0.03 | 1.16 | 0.070 |
|  | missing | 0.11 | 0.00 | 3.19 | 0.201 |
| Factor XII | 1^st^ | 1.00 |  |  |  |
|  | 2^nd^ | 1.73 | 0.33 | 8.94 | 0.514 |
|  | 3^rd^ | 3.41 | 0.57 | 20.41 | 0.179 |
|  | 4^th^ | 0.72 | 0.09 | 5.70 | 0.759 |
|  | 5^th^ | 1.37 | 0.11 | 16.44 | 0.804 |
|  | missing | 0.00 | 0.00 | . | 0.992 |
| Prothrombin | 1^st^ | 1.00 |  |  |  |
|  | 2^nd^ | 1.30 | 0.22 | 7.74 | 0.770 |
|  | 3^rd^ | 0.47 | 0.07 | 3.24 | 0.440 |
|  | 4^th^ | 1.96 | 0.26 | 14.97 | 0.518 |
|  | 5^th^ | 2.56 | 0.25 | 26.61 | 0.432 |
|  | missing | 53.96 | 0.02 | 92.38 | 0.992 |
| Factor XIII | 1^st^ | 1.00 |  |  |  |
|  | 2^nd^ | 0.41 | 0.10 | 1.73 | 0.224 |
|  | 3^rd^ | 0.31 | 0.07 | 1.28 | 0.106 |
|  | 4^th^ | 0.30 | 0.06 | 1.46 | 0.135 |
|  | 5^th^ | 1.20 | 0.26 | 5.57 | 0.814 |
|  | missing | 1.04 | 0.04 | 31.05 | 0.980 |

OR=Odds ratio, CI=Confidence Interval

**Figure 1 -** Accuracy comparison between clinical (dotted line) and clinical plus coagulation biomarker (solid line) prediction of in-hospital mortality among COVID-19 patients.
